# Imaging the Dynamics of the Electron Ionization of C_2_F_6_

**DOI:** 10.1021/acs.jpca.2c05606

**Published:** 2022-10-04

**Authors:** Patrick A. Robertson, David Heathcote, Dennis Milešević, Claire Vallance

**Affiliations:** Chemistry Research Laboratory, University of Oxford, OxfordOX1 3TA, U.K.

## Abstract

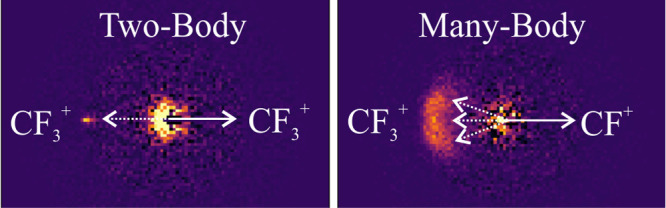

The dissociation of C_2_F_6_ following electron ionization at 100 eV has been studied using multimass velocity-map ion imaging and covariance-map imaging analysis. Single ionization events form parent C_2_F_6_^+^ cations in an ensemble of electronic states, which follow a multiplex of relaxation pathways to eventually dissociate into ionic and neutral fragment products. We observe CF_3_^+^, CF_2_^+^, CF^+^, C^+^, F^+^, C_2_F_5_^+^, C_2_F_4_^+^, C_2_F_2_^+^, and C_2_F^+^ ions, all of which can reasonably be formed from singly charged parent ions. Dissociation along the C–C bond typically forms slow-moving, internally excited products, whereas C–F bond cleavage is rapid and impulsive. Dissociation from the Ã state of the cation preferentially forms C_2_F_5_^+^ and neutral F along a purely repulsive surface. No other electronic state of the ion will form this product pair at the electron energies studied in this work, nor do we observe any crossing onto this surface from higher-lying states of the parent ion. Multiply charged dissociative pathways are also explored, and we note characteristic high kinetic energy release channels due to Coulombic repulsion between charged fragments. The most abundant ion pair we observe is (CF_2_^+^, CF^+^), and we also observe ion pair signals in the covariance maps associated with almost all possible C–C bond cleavage products as well as between F^+^ and each of CF_3_^+^, CF_2_^+^, CF^+^, and C^+^. No covariance between F^+^ and C_2_F_5_^+^ is observed, implying that any C_2_F_5_^+^ formed with F^+^ is unstable and undergoes secondary fragmentation. Dissociation of multiply charged parent ions occurs via a number of mechanisms, details of which are revealed by recoil-frame covariance-map imaging.

## Introduction

Electron ionization is a fundamental collision process that underpins the chemistry of a myriad of terrestrial and extraterrestrial environments.^1^ It is prevalent in the formation of plasmas,^2^ interstellar gas clouds,^3,4^ and terrestrial atmospheric processes^5,6^ and is at least partly responsible for radiative damage to biological tissue.^7^ Commonly, electron ionization is followed by fragmentation from either a dissociative state or a bound state of the parent molecular cation. The dynamics of these processes can influence the fragment product outcomes and can impact the ability to react further. Electron-ionization-driven chemistry has been shown to be important in gas-phase ion–molecule^4,5,8−10^ and dust-grain-surface-catalyzed reactions.^11^

Hexafluoroethane (C_2_F_6_) is widely used in the semiconductor fabrication industry as a dry-etching agent^12^ as well as in the enrichment of carbon-13.^13^ C_2_F_6_ is a potent greenhouse gas. It has an atmospheric lifetime greater than 2000 years^14^ and a 100 year global warming potential (GWP) of 11 500 (compared to CO_2_, which has a GWP of 1).^15^ Thus, the use and emission of C_2_F_6_, along with other perfluorocarbons, is strictly regulated under the Kyoto Protocol agreement.^16^ C_2_F_6_ in the ionosphere is routinely bombarded with high-energy electrons and photons, which can lead to molecular dissociation and subsequently the formation of ions and radicals.^17^ These dissociation products may go on to react further. To simulate the chemistry of the ionosphere of Earth and other planets, modelers rely on a multiplex of reaction kinetic information, including knowledge of secondary or tertiary byproducts of atmospheric chemical processes. Accurate modeling of C_2_F_6_ in the atmosphere requires understanding of the various dissociation channels available when the molecule is subjected to electron collisions.

The earliest reported dissociative ionization experiments on C_2_F_6_ were published by Bibby and Carter in 1963.^18^ As well as reported relative abundances of CF_3_^+^, C_2_F_5_^+^, CF_2_^+^, and CF^+^ ions following electron ionization at 35 eV, they also noted the formation of C_2_F_3_^+^ ions, which have not been observed in subsequent experiments. Lifshitz and Long reported relative abundances of these same product ions as well as appearance potentials for CF_3_^+^ and C_2_F_5_^+^ following 70 eV ionization of C_2_F_6_.^19^ These results were compared with Rice–Ramsperger–Kassel–Marcus (RRKM)^20^ calculations, which revealed that the C_2_F_5_^+^:CF_3_^+^ ratio was underpredicted by theory. Both of these early papers report the absence of a signal for the parent cation C_2_F_6_^+^, prompting Lifshitz and Long to suggest that the direct dissociation must occur from an electronically excited state of the cation, outcompeting redistribution of the excess electronic energy into vibrational motion.

This violation of the statistical theory generally used to describe the dissociation of molecular ions was confirmed by Simm et al., who recorded the photoelectron–photoion coincidence (PEPICO) spectrum of C_2_F_6_ following photoionization at 21.22 eV.^21−23^ Simm and co-workers showed that CF_3_^+^ product ions are exclusively formed from the ground electronic (X̃) state, whereas C_2_F_5_^+^ ions are formed from the first electronically excited (Ã) state of the cation. They also demonstrated that excited states above the Ã band (within the energy range studied) predominately form CF_3_^+^, providing clear evidence that the Ã state is isolated from any curve crossing points, and thus that parent ions formed in this state dissociate without any possibility of internal conversion.

Inghram et al. recorded breakdown curves for the C_2_F_6_^+^ ion in the energy range 14.14–18.64 eV using threshold PEPICO spectoscopy, reaffirming the existence of the isolated Ã state.^24^ They proposed that dissociation of the parent ion occurs on a time scale of less than 500 fs, comparable to the vibrational period of a C–F bond. The dynamics of dissociation was reported by Jarvis et al.^25^ using threshold PEPICO spectroscopy in the photon energy range 12–25 eV, confirming that the fragmentation was largely impulsive within this energy range.

The partial ionization cross sections for the formation of specific fragment ions of C_2_F_6_ following electron ionization have been reported numerous times in the literature,^26−30^ spanning energies from threshold up to 1000 eV. Among these reports, the most pertinent to this study is from the doctoral thesis of S.-J. King,^29^ who studied the dissociation of C_2_F_6_^*n*+^ (*n* = 1–3) following electron ionization between 30 and 200 eV via two-dimensional ion-coincidence spectroscopy. To our knowledge, this thesis reports the only study of the dynamics of the C_2_F_6_ dication (which accounts for almost 20% of total ion signal at 100 eV) and also reports partial ionization cross sections (30–200 eV) for single, double, and triple ionization events as well as coincident cross sections for the full suite of ion pairs and Monte Carlo-simulated total kinetic energy (KE) releases for dissociation of the parent di- and trication.

Doubly ionized molecules have emerged over the last two decades as potentially under-reported products of ionization events.^31−33^ Dications can generally be considered to be thermodynamically unstable in the gas phase. Their potential energy surfaces are often repulsive in nature because of many factors, including loss of bonding electrons as well as intramolecular Coulombic repulsion in charge-separated species. Dissociation of these highly energized ions is synonymous with high KE release in the daughter fragments. However, thermodynamically stable (e.g., OCS^2+^)^34^ and other metastable dication species are formed in multiple-ionization events, even for small molecules such as N_2_^2+^ and O_2_^2+^.^35^ Stable dications have been applied in ion–molecule collision experiments.^36−38^

Recently we have begun to explore the dissociation dynamics of multiply charged ions^35,39−42^ by using multimass velocity-map ion imaging (VMI) to record scattering distributions for all ionic products of electron ionization (EI) within a single measurement.^40,41^ The predominant outcome from an electron–molecule collision that leads to ionization is the formation of a singly charged parent ion. However, some proportion of collisions will create multiply charged ions via either an Auger cascade or a secondary collision of one of the departing electrons with another bound electron.^43,44^

For systems with multiple fragmentation pathways, it is a challenge to disentangle the dynamics of ions born from a multiply charged parent from that of ions formed from their singly charged counterpart. Any ion signal arising from fragmentation of a dication into two or more singly charged daughter ions invariably overlaps with signals arising from the dominant singly charged channels. Our approach to resolving the dynamics of multiply charged ions is to employ covariance analysis,^40−42,45,46^ a statistical method that reveals correlations between fragments even in the presence of much larger signals from uncorrelated events. The correlations of greatest interest to us are between product ion time-of-flight (TOF) spectra and product pair recoil velocities.

In the present work, we report results from a recent study of the electron-induced dissociation dynamics of C_2_F_6_ at an electron energy of 100 eV using an electron–molecule crossed-beam experiment with multimass velocity-map imaging detection. The data from these experiments provides insight into both singly and doubly charged dissociation channels within a single measurement, enabling a comprehensive exploration of the complex dynamics initiated by electron ionization of C_2_F_6_.

## Methods

### Experimental Section

The electron–molecule crossed-beam apparatus has been described in detail elsewhere.^46,47^ Briefly, a neat sample of C_2_F_6_ is pulsed into a high-vacuum chamber via a General Valve series 9 solenoid valve operating at 25 Hz. The resulting supersonic expansion is skimmed, and the skimmed molecular beam passes into the interaction region of a conventional velocity-map imaging ion optics arrangement^48^ interfaced with a TOF mass spectrometer. An electron gun (PSP Vacuum Technology, ELS100) outputs a 360 ns pulse of electrons with a kinetic energy of 100 eV (Δ*E* = 150 meV) that crosses the molecular beam at right angles. Once the electron beam passes through the interaction region, the repeller and extractor plates are rapidly switched from ground to velocity mapping potentials. Any ions formed are separated according to their mass to charge (*m*/*z*) ratio as they traverse the 240 mm flight tube before striking a position-sensitive detector (diameter = 40 mm) consisting of a pair of chevron-mounted microchannel plates and a P47 phosphor screen. The light emitted from the phosphor is imaged using a pixel-imaging mass spectrometry (PImMS) camera,^49^ which records (*x*, *y*, *t*) coordinates for each ion strike with 25 ns resolution. Signal and background data sets are recorded over 750 000 experimental cycles each. In the “background” cycles, the electron pulse is timed to arrive at the interaction region before the molecular beam, while in the “signal” cycles, the relative timings of the two pulses are adjusted for optimum overlap. The resulting data set can be integrated over the *x* and *y* coordinates to obtain a TOF mass spectrum, and two-dimensional crushed velocity-map images of the product scattering distributions for each detected ion are obtained by integrating over the appropriate arrival time intervals. TOF spectra are also obtained independently by measuring the total (time-dependent) signal from the phosphor screen using a photomultiplier tube coupled to an oscilloscope.

### Data Analysis

A centroiding algorithm^50^ is employed to reduce individual ion strikes to a single pixel in position and time. Centroided velocity-map images are symmetrized, and the central slice of the three-dimensional scattering distribution is obtained via an Abel inversion using the BASEX algorithm within the PyAbel package.^51^ An angular integration is then carried out to obtain radial distributions, which are then converted from pixels into kinetic energy using a velocity calibration determined via ion trajectory simulations performed in SIMION 8.0.^52^

Centroided images are also subjected to covariance analysis in order to identify correlated TOF and velocity distributions for pairs of ions. Briefly, covariance is a general statistical method that is used to determine correlations between two variables, in this instance, the arrival time or velocity of two ions of interest. True covariances are seen only between two product ions formed from the same parent ion, and thus, only dissociation events involving multiply charged ions will contribute to the covariance signal, i.e., the analysis is blind to signals arising from dissociation of singly charged ions, which account for a significant majority of the total signal. The principles of covariance in the context of mass spectrometry were originally outlined by Frasinski et al.^53^ for high-count-rate experiments, which are not compatible with coincidence measurements. More recently, covariance analysis has been applied to data from velocity-map imaging experiments.^40,41,54^ Mathematically, the covariance between two variables *X* and *Y* is defined as the average of the product of the deviations of these quantities from their respective mean values:
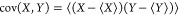
1
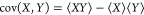
2where ⟨···⟩ denotes an average over experimental cycles. If an increase in *X* tends to correspond to an increase in *Y*, then the covariance will be positive. In our case, *X* and *Y* are either the arrival times (*t*) or the (*x*, *y*) coordinates of two ions.

To account for variation in signal due to fluctuating experimental parameters, we apply a corrected form of covariance, termed “partial covariance”, which is described in detail elsewhere.^40^ This correction is given by the following equation:
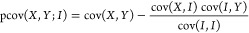
3where *I* is a variable that accounts for the varying experimental parameters such as molecular beam density and/or electron beam current. In the present work, rather than continuously monitoring the beam intensities, we use the fact that the signal depends linearly on both and take *I* to be the total ion signal recorded during each experimental cycle. In practice, we take a rolling average of the total ion signal over 20 s in order to smooth out shot-to-shot fluctuations.

In the present work, we utilize two forms of partial covariance: TOF–TOF covariance, which shows the correlation between the arrival times of different ions, and recoil-frame covariance, which correlates the relative velocity vectors of two ions.

## Results and Discussion

**Figure 1:**
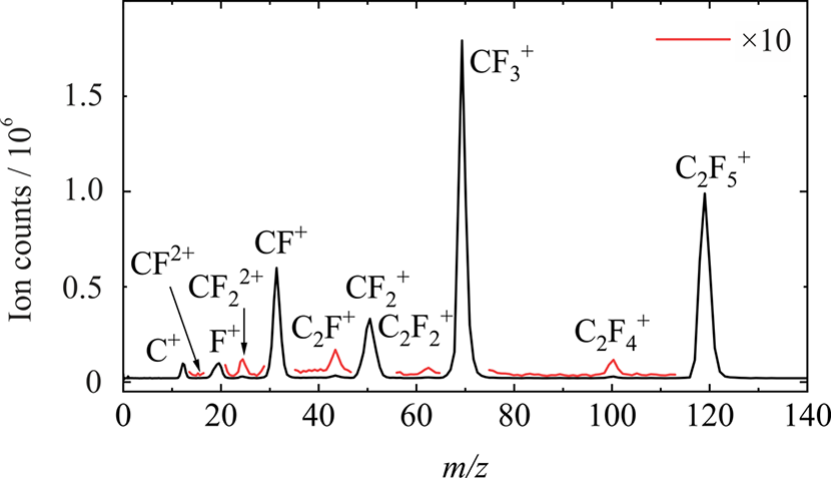
Time-of-flight mass spectrum for the products of 100 eV electron impact ionization of C_2_F_6_.

The TOF mass spectrum of C_2_F_6_ recorded at an electron energy of 100 eV is shown in Figure 1. The fragment signals observed are in reasonable agreement with those reported in the National Institute of Standards and Technology electron ionization database at 75 eV^55^ as well as those reported by King for ionization at 200 eV.^29^ The most abundant fragments are CF_3_^+^ and C_2_F_5_^+^, followed by CF^+^ and then CF_2_^+^. We see no signal attributable to intact parent C_2_F_6_^+^ cation. We do see subtle yet clear evidence for the formation of C_2_F_4_^+^, C_2_F_2_^+^, and C_2_F^+^ as well as the doubly charged fragments CF_2_^2+^ and CF^2+^. Any potential signal arising from CF_3_^2+^ (*m*/*z* = 34.5) appears to be masked by the CF^+^ (*m*/*z* = 31) peak. The doubly charged ions CF_3_^2+^ and CF_2_^2+^ are known to be stable on the microsecond time scale of our experiments and are readily formed from double ionization of CF_4_.^32,37^

### Velocity-Map Ion Images

**Figure 2:**
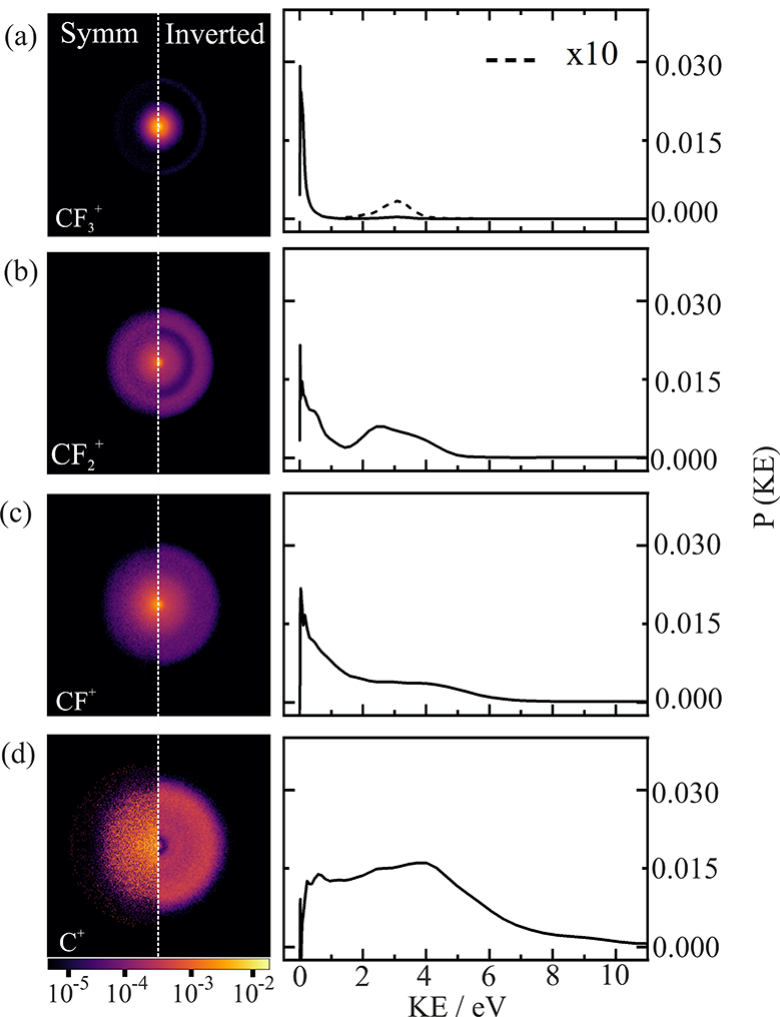
Symmetrized and inverted scattering distributions for (a) CF_3_^+^, (b) CF_2_^+^, (c) CF^+^, and (d) C^+^ following 100 eV electron ionization and the corresponding kinetic energy distributions for each fragment product. Image intensities are plotted on a logarithmic scale to assist in visualizing weaker features.

Figure 2 shows symmetrized and Abel-inverted velocity-map images for the CF_*n*_^+^ ions (C^+^, CF^+^, CF_2_^+^, and CF_3_^+^), together with their corresponding kinetic energy distributions. The KE plots show a generally bimodal distribution; each fragment (except C^+^) features a low-KE-release channel peaking at 0 eV as well as a higher-KE component attributable to dissociation of a multiply ionized parent molecule. The high-KE channel increases in intensity relative to the low-KE peak as the CF_*n*_^+^ fragment loses F atoms. Fragmentation from multiply charged parent ions will be discussed in detail separately in a later section, and we will focus for now on the dissociation products of singly charged parent ions.

Electron ionization at the energies of interest can be considered a ballistic process. An incident electron collides with a bound electron within the molecule, leading to ionization and the formation of an electron hole. The vast majority of the collision energy is carried away by the departing electrons, and the very short time scale (tens to hundreds of attoseconds) of the encounter means that little or no energy is tranferred into the nuclear framework during the collision. The subsequent nuclear dynamics is therefore governed by the response of the nuclear framework to the sudden appearance of the electron hole formed in the collision.^56,57^

The low-KE Boltzmann-like distributions of the CF_*n*_^+^ ions are indicative of the dissociation of a highly vibrationally excited parent ion in a bound or metastable electronic state.^56^ These dissociative processes typically occur over a relatively long time scale, such that geometric relaxation and vibrational energy transfer are competitive with dissociation. This allows statistical distribution of energy over the energetically accessible internal vibrational states of the cation prior to dissociation,^58^ with dissociation occurring once sufficient energy becomes available in a mode coupled to the appropriate reaction coordinate.

The low-energy component of the KE distribution becomes broader, extending to higher KE, as the number of F atoms remaining on the carbon atom decreases. This is attributable to the loss of neutral fluorine atoms in concert with the dissociation of the carbon–carbon bond. If the C–F bond breaks prior to the C–C bond, then as a result of the reduction in mass, the observed CF_*n*_^+^ (*n* < 3) fragment is likely to be born with a broader velocity (and therefore KE) distribution, peaking at somewhat higher velocities. The formation of vibrationally excited CF_*n*_^+^ fragments can also result in the loss of fluorine. Impulsive dissociation along the C–C bond axis may excite vibrational motion in the departing fragments and subsequent loss of one or more F atoms.

**Figure 3:**
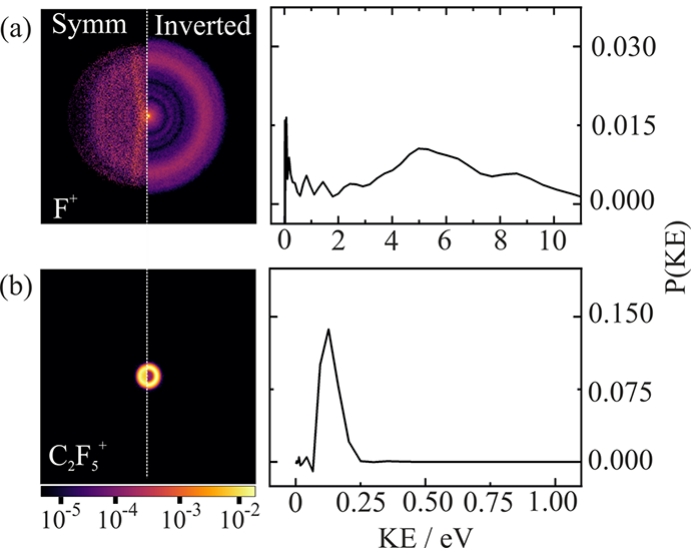
Symmetrized and inverted scattering distributions for (a) F^+^ and (b) C_2_F_5_^+^ following 100 eV ionization and the corresponding kinetic energy distributions for each fragment product. Image intensities are plotted on a logarithmic scale.

Figure 3 shows symmetrized and Abel-inverted velocity-map images of F^+^ and C_2_F_5_^+^, accompanied by their respective KE distributions. Fluorine ion (*m*/*z* = 19) images contain appreciable contamination from background water (*m*/*z* = 18) in our experimental apparatus, but this is localized largely along the center of the image along the electron beam path. Despite this contamination, a broad isotropic scattering distribution peaking at high KE (around 5 eV) can be clearly resolved. The appearance energy for the formation of F^+^ from C_2_F_6_ is around 35 eV, as reported by Iga et al.,^27^ and approximately coincides with the onset of double ionization, as reported by King.^29^ The breadth of the KE distribution perhaps implies the formation of F^+^ via a number of dissociation channels, but the high KEs indicate that all involve multiply charged parent ions. We therefore defer any further discussion of F^+^ formation to the later sections in which we probe such channels via covariance analysis.

Loss of neutral F atom leads to the formation of C_2_F_5_^+^ ions with kinetic energy peaking away from zero, at 0.13 eV. This is characteristic of prompt dissociation from a repulsive surface rather than the more Boltzmann-like statistical kinetic energy distribution we observe for breaking of the C–C bond of the singly charged parent ion. Stockbauer and co-workers^24^ noted that the onset energy for the formation of C_2_F_5_^+^ ion is within the experimental uncertainty of the onset of the Ã state of the ion (17.5 eV). The observed nonstatistical KE release is consistent with the formation of C_2_F_5_^+^ directly from the Ã state without internal conversion. Simm et al.^22^ assigned this state to ionization of a lone-pair orbital on the fluorine atom, creating a vacancy that is rapidly refilled by electron transfer from a C–F bonding orbital, ultimately leading to C–F bond cleavage and the formation of stable C_2_F_5_^+^ and neutral fluorine. Photoionization studies on C_2_F_6_ show a very narrow window for the formation of C_2_F_5_^+^ ions from approximaitely 15 to 18 eV.^21,22,24^ Despite this narrowly accessible energy range, C_2_F_5_^+^ is our second most abundant fragment product because of the sixfold degeneracy of the C–F bond. Outside of this range, C–C bond cleavage is the overwhelmingly dominant process. We observe no other contributing channel to the formation of C_2_F_5_^+^, supporting the hypothesis that the Ã state of the parent ion is entirely isolated from any crossing point, at least at the energies accessed in the present study.

The fragment products C_2_F_4_^+^, C_2_F_2_^+^, and C_2_F^+^ do not exhibit the same purely impulsive dissociation dynamics as C_2_F_5_^+^ (see the Supporting Information). C_2_F_4_^+^ has a close to statistical KE distribution centered around 0 eV, whereas C_2_F_2_^+^ and C_2_F^+^ show a mixture of both low- and high-KE channels, synonymous with formation from multiple ionization states of the parent molecule. King reported that up to 90% of C_2_F_*n*_^+^ (*n* = 1, 2, 4) is formed from singly charged parent ion at 100 eV,^29^ whereas singly charged parent ions account for only 80% of the ion yield at this energy.

### Time-of-Flight Covariance Maps

**Figure 4:**
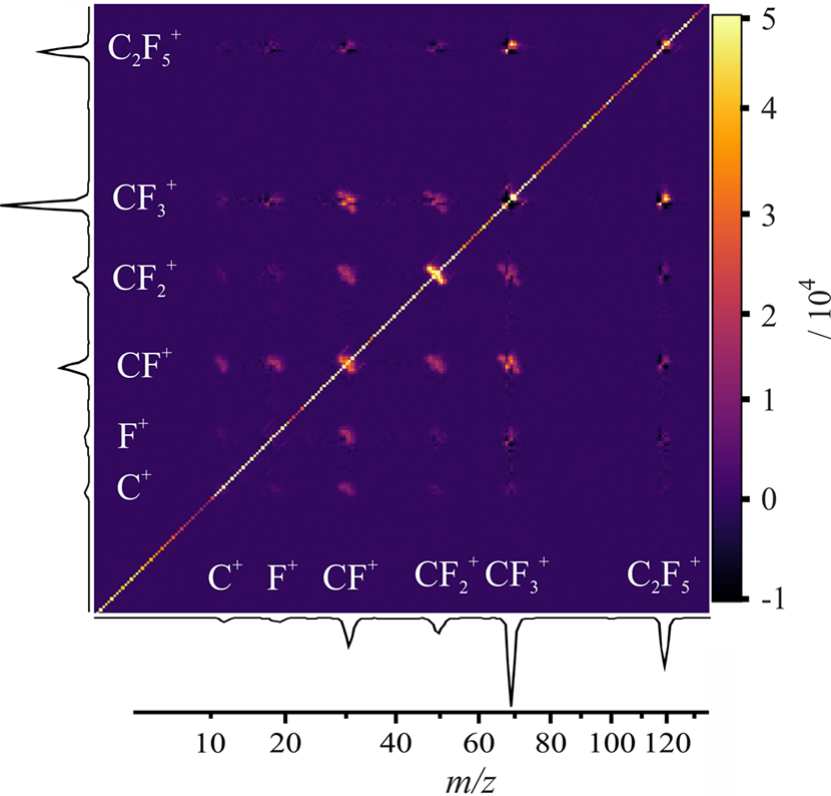
TOF–TOF covariance map for the dissociative electron ionization products of C_2_F_6_ recorded at an electron energy of 100 eV.

We now move on to consider fragmentation pathways involving multiply charged parent ions. Figure 4 shows the TOF–TOF partial covariance map for the products of 100 eV electron ionization of C_2_F_6_. The signal along the diagonal corresponds to the variance of the TOF spectrum, and off-diagonal elements indicate covariances between arrival times of various ions. The gradient of off-diagonal features is determined by the ion momenta and therefore depends on the ion masses and charges and the mechanism of dissociation, as discussed in detail previously.^41,59,60^ For example, two-body dissociations (e.g., CF_3_^+^ + CF_3_^+^) are characterized by a slope of −1 as a result of conservation of momentum. In this case the two ions will have equal and opposite momentum components along the TOF axis, with one ion arriving slightly earlier than the peak in the arrival time distribution for that ion and the other arriving slightly later. This difference in arrival time is proportional to the ion momenta along the TOF axis, and this yields a gradient of −1 for all of the matched pairs in the covariance map. More complex many-body mechanisms change or blur the gradient of any off-diagonal feature. We also observe a number of small signals corresponding to impossible (or “false”) covariances, for example, between C_2_F_5_^+^ and various CF_*n*_^+^ fragments, which are the result of imperfect performance of the partial covariance correction. False covariances are characterized by an off-diagonal postive covariance feature with slope of +1, often surrounded by a negative covariance signal along the slope of −1, whereas true covariances appear as off-diagonal signals with positive intensity and a negative gradient. False covariance may also be confirmed by checking the corresponding recoil-frame covariance maps, as discussed later.

We observe many “true” positive covariance features between ions formed via C–C bond cleavage. In agreement with King,^29^ the most intense product ion pair is (CF_2_^+^, CF_2_^+^), followed by (CF_3_^+^, CF^+^). We see covariances between all C–C bond cleavage products at 100 eV. Based on the observed gradients of the covariance signals, these dissociations all appear to be “pseudo-two-body” in mechanism, with the departing F atom recoil imparting a less significant momentum “kick” than the charge separation step.

F^+^ covariances include the ion pairs (F^+^, CF^+^) and (F^+^, CF_2_^+^) as well as weaker features (see the Supporting Information) corresponding to (F^+^, CF_3_^+^), (F^+^, C_2_F^+^), (F^+^, CF_2_^2+^), and (F^+^, C^+^). We see no identifiable signal from the (F^+^, C_2_F_5_^+^) ion pair. A rationalization for this null result is discussed in detail later.

While it is possible to elucidate some details of the dissociation mechanisms from the TOF–TOF covariance map, our relatively low TOF resolution limits the extent to which we are able to determine the gradient of signals arising from more complicated dissociation channels. In the following section, we will demonstrate that covariances between ion velocities, in the form of recoil-frame covariance maps, produce more sophisticated and intuitive insights into the mechanism of dissociation.

### Recoil-Frame Covariance Maps

**Figure 5:**
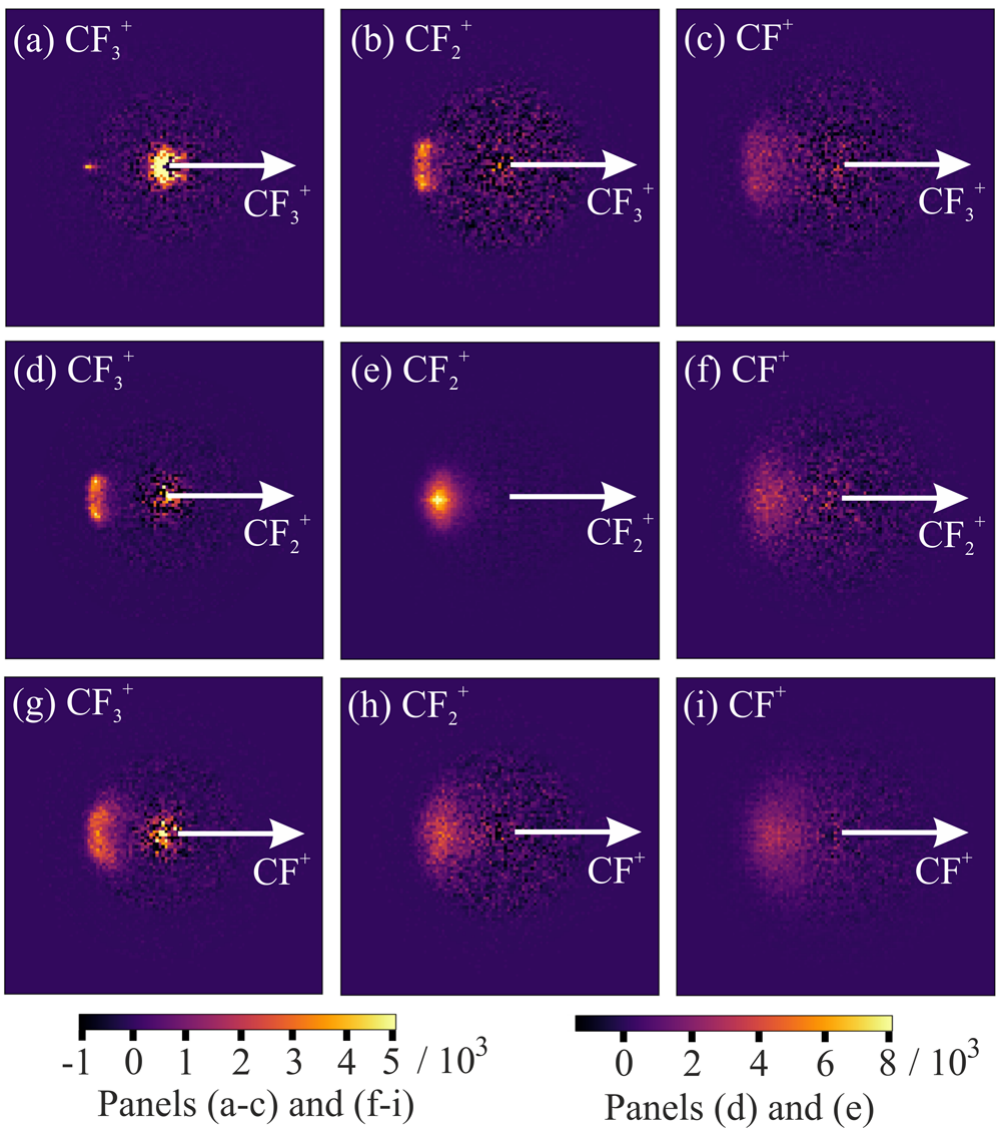
Recoil-frame partial covariance images for the ion pairs (S^+^, R^+^) formed via C–C bond cleavage in the 100 eV dissociative electron ionization of C_2_F_6_, where S^+^ is the signal ion and R^+^ is the reference ion: (a) (CF_3_^+^, CF_3_^+^); (b) (CF_2_^+^, CF_3_^+^); (c) (CF^+^, CF_3_^+^); (d) (CF_3_^+^, CF_2_^+^); (e) (CF_2_^+^, CF_2_^+^); (f) (CF^+^, CF_2_^+^); (g) (CF_3_^+^, CF^+^); (h) (CF_2_^+^, CF^+^); (i) (CF^+^, CF^+^). The reference direction is indicated by the white arrow in each case. In images (a), (e), and (i), the self-covariance signal has been removed by masking the covariance signal over an angle of 1° around the reference direction. All of the images have been normalized such that the positive covariance sums to unity.

Figure 5 shows the recoil-frame partial covariance maps for ion pairs formed from the dissociation of multiply ionized C_2_F_6_ along the C–C bond coordinate. For each ion pair (A^+^, B^+^), we assign one of the ions to be the “signal” ion and the other to be the “reference” ion. The reference ion velocity is constrained to lie along the positive *x* axis, indicated by the white arrows in Figure 5, and the covariance map shows the directions in which signal ions are scattered relative to this reference direction. For each ion pair, we display both permutations of the signal and reference. Figure 5a shows the covariance map for the (CF_3_^+^, CF_3_^+^) ion pair. This ion pair is formed via a simple two-body dissociation, in which conservation of momentum requires that the two products recoil in opposite directions with equal and opposite momenta. This results in a covariance map in which the covariance signal from the signal ion appears as a well-defined spot along the reference axis with a radius equal to that of the outer ring in the corresponding velocity map image (see Figure 2, image (a)). The center of image (a) in Figure 5 contains considerable noise due to false covariances arising from CF_3_^+^ ions formed from singly charged parent ions within the same experimental cycle, which are formed in such large quantities that they are imperfectly canceled by the partial covariance correction.

For more complex dissociation channels, recoil-frame covariance maps contain a great deal of information on the various multistep mechanisms that lead to a pair of products. As we look from images (a) through (c) of Figure 5, the relative velocity distribution of the CF_*n*_^+^ signal ion progressively blurs. This is a consequence of the momentum “kick” imparted to the products during C–F bond fission, which reduces the correlation between the relative velocities of the two fragment ions. Three general mechanisms for dication dissociation can be defined according to the order in which the charge separation step (C) occurs relative to any neutral-loss steps (n). For the dissociation pathway yielding the (CF_2_^+^, CF_3_^+^) ion pair, the possible mechanisms are the following:concerted dissociation:
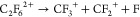
initial charge separation (Cn):
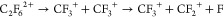
deferred charge separation (nC):
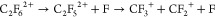
These processes, which represent limiting cases for a two-step dissociation, result in characteristic recoil-frame scattering distributions.^41^ The form of these distributions depends on both the sequence in which the charge separation step occurs relative to the neutral dissociation and the relative masses of the various fragments. The formation of some of the observed ions involves additional F-loss steps, but we start by addressing the two-step pathways.

King proposed that dissociation proceeds via the “fast sequential” deferred charge separation (nC) mechanism in his model for the two-step breakup of C_2_F_6_^2+^, with the initial F atom loss occurring on a much shorter time scale than the subsequent charge separation step.^29^ For example:
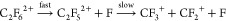
This mechanism is consistent with the postulate of Inghram et al.^24^ that the C–F bond cleavage occurs on the femtosecond time scale whereas the C–C bond cleavage is much less prompt. In such a mechanism, the recoil blurring imposed on the C_2_F_5_^2+^ fragment as a result of F atom loss is small due to the imbalance in mass of the two fragments, and the charge separation step dominates the relative velocity distribution of the product ion pair. This mechanism also helps to explain the relatively well defined signal-ion scattering distribution for the (CF_3_^+^, CF_2_^+^) ion pair (see panels (b) and (d) in Figure 5). The distribution matches qualitatively with simulated covariance maps corresponding to the nC mechanisms in CF_3_I^2+^ reported previously.^41,46^ In such a mechanism, the recoil velocities of both CF_*n*_^+^ fragments include the momentum kick from the C–F bond cleavage, whereas in an initial charge separation (Cn) mechanism the trajectory of only one of the ions is affected by the C–F bond dissociation.

As the dissociation of the parent dication becomes increasingly multistep in nature, the relative velocity distributions become increasingly more blurred, as the C–F bond cleavage momentum kicks account for an increasingly greater proportion of the total momentum of the covariant CF_*n*_^+^ fragments.

**Figure 6:**
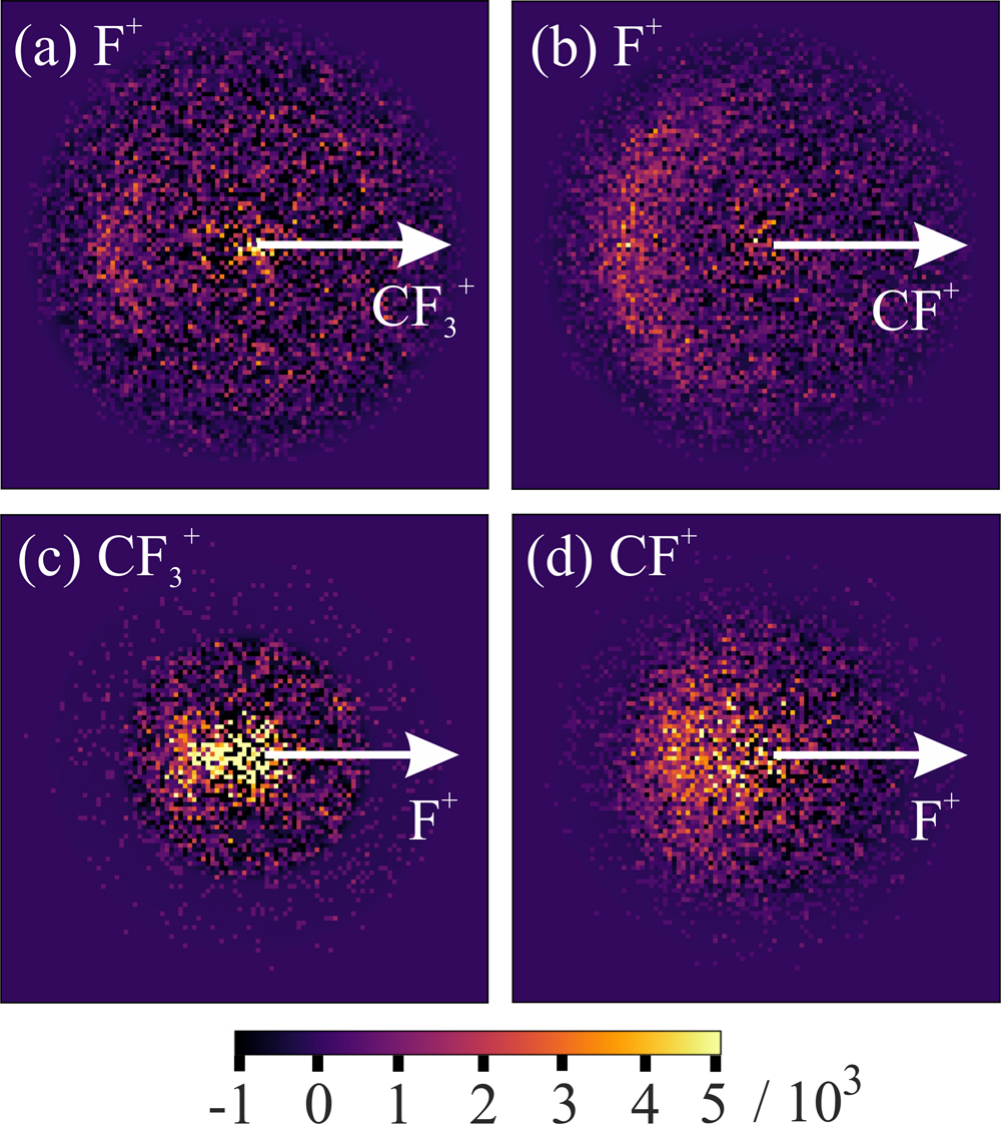
Recoil-frame partial covariance images for the ion pairs (S^+^, R^+^), where S^+^ is the signal ion and R^+^ is the reference ion, formed via dissociation pathways involving both C–C and C–F bond cleavage in the 100 eV dissociative electron ionization of C_2_F_6_: (a) (F^+^, CF_3_^+^); (b) (F^+^, CF^+^); (c) (CF_3_^+^, F^+^); (d) (CF^+^, F^+^). The reference direction is indicated by the white arrow in each image, and all of the images have been normalized such that the positive covariance sums to unity.

Figure 6 shows recoil-frame covariance maps for the ion pairs (F^+^, CF_3_^+^) and (F^+^, CF^+^). In each of these covariance maps, we see a well-defined arc of F^+^ signal ions, more characteristic of a Cn mechanism. If F^+^ loss from C_2_F_6_^2+^ outpaces C–C bond cleavage, then we would expect a well-defined recoil velocity of F^+^, as its trajectory is established prior to the secondary dissociation step(s) that form either CF_3_^+^ or CF^+^. On the basis of coincidence measurements,^29^ King proposed a general mechanism by which the C_2_F_6_^2+^ dication dissociates to form all (F^+^, CF_*n*_^+^) ion pairs. In agreement with our observations, King suggested that F^+^ is rapidly lost, followed by decay of the remaining C_2_F_5_^+^ moiety. Given that we see no covariance between F^+^ and C_2_F_5_^+^, we must assume that all pathways for the dication to form F^+^ must produce an unstable C_2_F_5_^+^ fragment. This is consistent with our single-component KE distribution for the C_2_F_5_^+^ fragment, which is stable only when formed from the Ã state of the monocation.^22^

## Conclusions

We have presented a comprehensive experimental study of the dissociative electron ionization dynamics of C_2_F_6_ at an electron energy of 100 eV. We see contributions from singly and multiply charged parent cations, which dissociate into a wide variety of C–C and C–F bond cleavage products. Multimass velocity-map ion images and the corresponding kinetic energy distributions for each fragment provide mechanistic insight into the dissocation dynamics. Covariance analysis allows us to explore the dissociation dynamics of the multiply charged parent ions, exploiting the wealth of information contained within multimass imaging data sets.

Focusing on the singly charged channels first, we observe a single dissociative pathway to the formation of C_2_F_5_^+^ via the Ã state of the parent cation. This dissociation is rapid and impulsive, outcompeting internal conversion and forming neutral F and a C_2_F_5_^+^ ion with a product ion kinetic energy distribution centered around 0.13 eV. In contrast, we observe Boltzmann-like statistical distributions of kinetic energy for all CF_*n*_^+^ (*n* = 1–3) products, which peak at 0 eV and extend out beyond 1 eV. We assign this statistical distribution to a number of different pathways:

1. Parent C_2_F_6_^+^ cations are initially formed in a number of electronic states that relax to a subset of lower-lying states via internal conversion and/or radiative decay. Cations in these lower-lying states then dissociate on relatively long time scales following statistical redistribution of internal vibrational energy to give CF_3_^+^, CF_2_^+^, and CF^+^ ions.
2. Neutral fluorine loss from C_2_F_6_^+^ typically precedes the dissociation of the resulting C_2_F_*n*_^+^ ion into CF_*n*_^+^ product ions.

Covariance-map imaging is used to study dissociation pathways of multiply charged parent ions formed in electron–molecule collisions. TOF–TOF covariance maps reveal that all possible CF_*n*_^+^ ion pairs are observed. Covariance signals are also seen between F^+^ and C^+^, CF^+^, CF_2_^+^, CF_3_^+^, C_2_F^+^, and CF_2_ but not between F^+^ and C_2_F_5_^+^.

Recoil-frame covariance maps allow us to explore the complex multistep dissociation mechanisms of C_2_F_6_^2+^ dications. Based on these covariance maps, we propose plausible unimolecular reaction mechanisms. Reactions that form two CF_*n*_^+^ ions are thought to proceed via a deferred charge separation mechanism, in which the loss of neutral fluorine occurs rapidly, imparting only a small kick to the remaining C_2_F_5_^2+^ dication. This dication then dissociates with a kinetic energy release upward of 3 eV resulting predominately from Coulombic repulsion between the two charges. Channels forming (F^+^, CF_*n*_^+^) ion pairs follow an initial charge separation mechanism in which F^+^ departs rapidly and the remaining monocation then undergoes further dissociation into an ion–neutral pair.

This work highlights the power of multimass ion imaging and covariance-map imaging in revealing detailed mechanistic information on the dissociation dynamics of multiply charged ions. This is possible even in the presence of much larger signals arising from the dissociation of singly charged ions. Information on the full range of fragment channels can be obtained in a single measurement under high-count-rate conditions, offering a user-friendly alternative to conventional coincidence measurements for understanding chemical dynamics.

## Supporting Information Available

Velocity-map ion images and kinetic energy distributions for fragment products CF_2_^2+^, C_2_F^+^, C_2_F_2_^+^, and C_2_F_4_^+^ and TOF–TOF covariance map with increased contrast to resolve weaker covariance features (PDF)
