# Supplementary material for: Imaging the Dynamics of the Electron Ionization of C2F6
Source: J Phys Chem A. 2022 Oct 4;126(40):7221–9. doi: 10.1021/acs.jpca.2c05606 (PMC9574930; doi:10.1021/acs.jpca.2c05606)
Supplement: Supplementary file 1 — jp2c05606_si_001.pdf [file jp2c05606_si_001.pdf]

Supporting Info for Imaging the Dynamics of the Electron Ionisation of C_2F_6

Patrick A. Robertson, David Heathcote, Dennis Milešević, and Claire Vallance*

Chemistry Research Laboratory, University of Oxford, Oxford, UK, OX1 3TA

E-mail: claire.vallance@chem.ox.ac.uk

Additional Figures

Velocity-Map Images

Figure S1 shows symmetrised and Abel inverted velocity-map images for fragment ions CF_2^{2+}, C_2F^+, $C_2F_2^+$, and $C_2F_4^+$ and their corresponding kinetic energy distributions

TOF-TOF covariance

High contrast TOF-TOF covariance maps are shown in figure S2 and reveal more subtle covariance features. Of note are covariances between C_2F^+ and F^+, as well as between CF_2^{2+} and F^+. The latter is direct evidence of triply ionised parent C_2F_6.

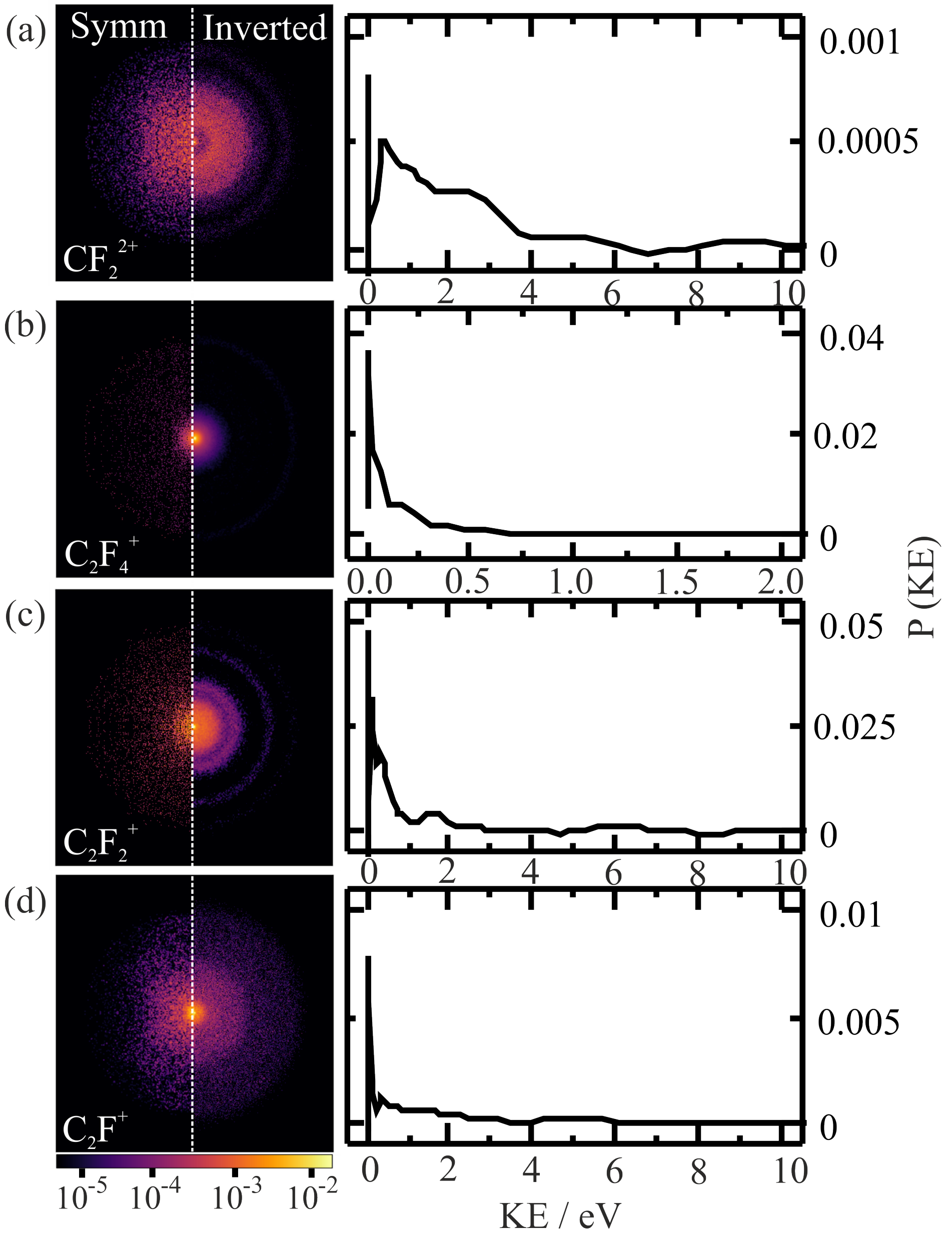

Figure S1: Symmetrised and inverted scattering distributions of C_2F^+, $C_2F_2^+$, $C_2F_4^+$, CF_2^{2+} following 100 eV electron ionisation, and corresponding kinetic energy distributions for each fragment product. Image intensities are plotted on a logarithmic scale to help resolve weaker features

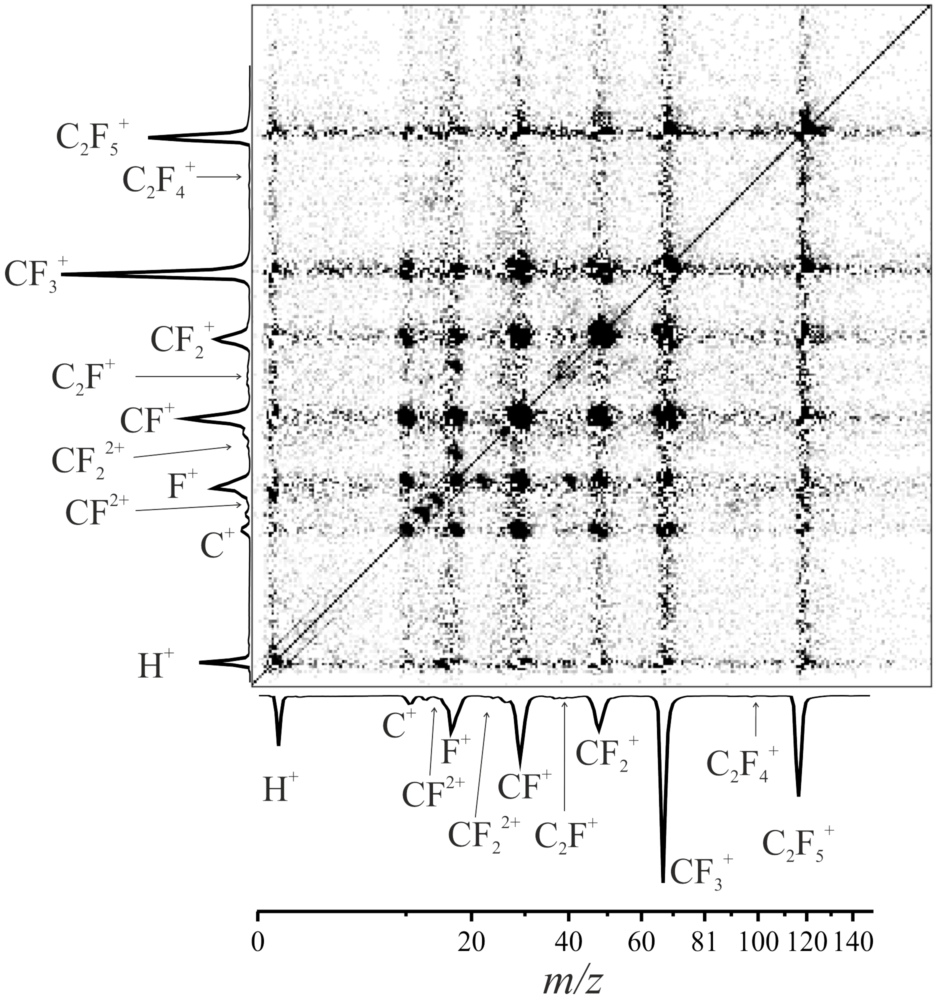

Figure S2: High contrast Time-of-Flight - Time-of-Flight (TOF-TOF) covariance map of C_2F_6 recorded at an electron energy of 100 eV.
